# Catalogue of the Human Crania in the Collection of the Academy of Natural Sciences of Philadelphia

**Published:** 1857-07

**Authors:** 


ART. VII.—*Catalogue of the Human Crania in the Collection of the Academy of Natural Sciences of Philadelphia.* Based upon the Third Edition of Dr. Morton's "Catalogue of Skulls." By J. AITKEN MEIGS, M.D., Librarian of the Academy, &c. Pamphlet, pp. 112. J. B. Lippincott & Co., Philadelphia, 1857.

DR. MEIGS deserves the thanks of all who are interested in the subject of cranioscopy, for his efficient labor in arranging the large collection of skulls in the Academy of Natural Sciences, and for the equally efficient manner in which he has succeeded in framing a catalogue of the same. The work is not, however, simply a list of the various specimens, but is more or less descriptive, and illustrated by numerous wood-cuts, which render it of essential service to persons at a distance. The following extract from the "explanatory note," prefixed to the Catalogue, will give our readers some idea of the character and extent of this magnificent collection:

Since the death of the late lamented President of the Academy of Natural Sciences,—DR. SAMUEL GEORGE MORTON,—his magnificent Collection of Human Crania, recently increased by the receipt of 67 skulls from various sources, has been permanently deposited in the Museum of the Academy. Prior to his demise, Dr. M. had received 100 crania in addition to those mentioned in the third edition of his Catalogue. Since 1849, therefore, the collection has been augmented by the addition of 167 skulls. To complete the Catalogue in a uniform manner, these have been carefully numbered and measured in accordance with the methods recorded in the *Crania Americana*, &c. In a portion of these measurements I was kindly assisted by our fellow-member, Dr. Thomas J. Turner, of the United States Navy.

The entire collection, numbering 1035 crania, was purchased by forty-two gentlemen from the executors of Dr. Morton, for the sum of $4000, and by them generously presented to the Academy.

The collection occupies sixteen cases on the first gallery, on the south side of the lower room of the Museum. For convenience of study and examination, I have grouped the crania according to race, family, tribe, &c., strictly adhering, however, to the classification of Dr. Morton. It will be seen, also, that the same arrangement has been adopted in this edition of the Catalogue, so that it is an exact representation of the collection as it stands upon the shelves. While the old numbering has been carefully preserved for the sake of reference to the various published descriptions of Dr. Morton, new numbers have been added to designate the position of any skull in the natural division or subdivision to which it belongs.

The Suevic, Cimbric, and Scandinavian divisions of the great Teutonic race, are represented by 32 crania and three casts, distributed as follows: 1 Norwegian, 7 Swedish peasants, 2 Swedes from Finland, 3 Swedes from Sudermanland, 1 Ostrogoth, 1 Turannic Swede, 2 Cimbric Swedes, 1 Cimbrian from Moën Island, 11 Germans, 1 Dutchman, 4 Prussians, and 1 Ancient Burgundian. Among these I have also placed 3 Swedish Finns, which, though mixed, are more Swedish than Finnish. Next to these, from their affinity, have been arranged the heads of 9 true Finns, and a cast of a Finlander's skull.

Of 4 Swedish peasants, the highest internal capacity is 99, the lowest 65, while the average of the group is 83 cubic inches. Of 2 Swedes from Finland, the larger is 107·5, the smaller 93·75, and the mean 100·62. Of 3 Swedes from Sudermanland, the highest measurement gives 108·25, the lowest 102, and the mean 101·41 inches. Of 2 Cimbric Swedes, the higher is 94, the lower 80, the mean 87. Of 10 German heads, the highest is 104, the lowest 70, and the mean of the series 88·6. The skull of a Dutch gentleman (No. 434), is the largest in the entire collection, for it measures 114 cubic inches of internal capacity. 4 Prussian skulls give 92 for the highest, 80 for the lowest, and 83·5 for the mean. The average for this branch of the Teutonic family, as deduced from the foregoing measurements is about 94 cubic inches.

Of 3 Swedish Finns, the highest internal capacity is 89, the lowest 85, and the mean 87 inches. Of 9 true Finns, the highest is 113·5, the lowest 81·5, the mean 94·3. A large proportion of this valuable series, from Nos. 1545 to 1550, and from 1542 to 1541, were received from Professor Retzius, after the death of Dr. Morton.

